# Per- and polyfluoroalkyl substances exposure and endometriosis risk: Evidence from epidemiologic, network toxicology, and molecular docking

**DOI:** 10.1016/j.isci.2025.114145

**Published:** 2025-11-19

**Authors:** Haiqiao Zhang, Siliang Zeng, Jingyun Yu, Fengming Zhu, Xiandan Yang, Weichao He, Yuwei Liu, Yu Liang, Yang Liang, Wenxin Hong, Qian Yuan

**Affiliations:** 1 Department of Health, Dongguan Maternal and Child Health Care Hospital, Dongguan 523000, China

**Keywords:** biochemistry, environmental toxicology, molecular toxicology

## Abstract

Although per- and polyfluoroalkyl substances (PFAS) exposure has been linked to endometriosis, this association remains controversial, and the underlying mechanisms are unclear. This study aimed to investigate this relationship and explore its molecular basis. Using cross-sectional data from NHANES, we analyzed serum PFAS in 1,069 women (20–50 years), applying WQS and BKMR models to assess mixture effects. Network toxicology (protein-protein interaction, pathway enrichment), molecular docking, and external validation were also used. Results showed PFAS mixtures were positively associated with endometriosis (adjusted OR = 1.22, 95% CI: 1.08–1.39), with PFOA and PFOS as main contributors. Mechanistic analysis revealed 129 overlapping genes involved in steroid hormone signaling, inflammatory responses, and the PI3K-Akt pathway, along with potential disruptions in lipid metabolism and oxidative stress. This work provides epidemiological and mechanistic evidence that PFAS mixtures may promote endometriosis via endocrine disruption and inflammatory activation, highlighting the need for further research into their gynecological health effects.

## Highlights

• NHANES links PFAS mixtures to endometriosis risk; PFOA/PFOS are key drivers
• Network analysis finds 129 shared genes in hormone/inflammation/PI3K-Akt pathways
• Molecular docking confirms PFAS bind core targets, implicating endocrine disruption
• Multi-model approach reveals PFAS mixture synergy, advancing toxicity assessment

## Graphical abstract

**Figure:**
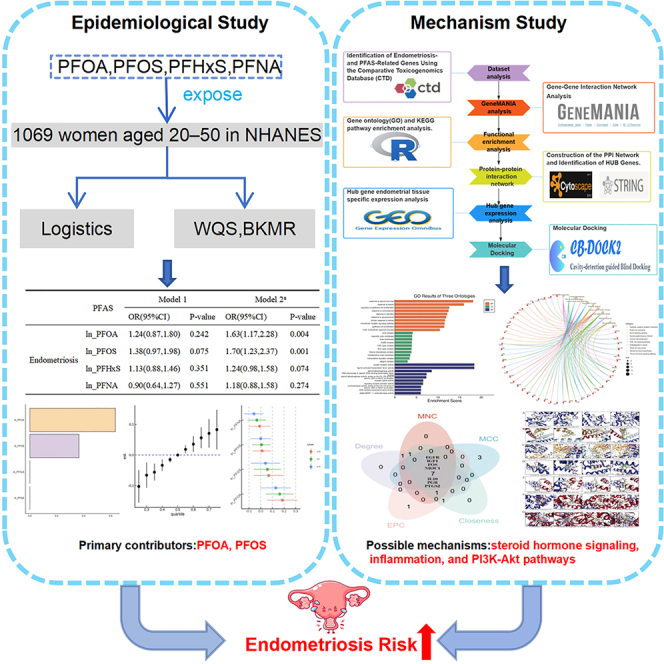

## Introduction

Per- and polyfluoroalkyl substances (PFASs) are a group of persistent organic pollutants that continue to present global environmental and public health concerns due to their extreme persistence and documented bioaccumulation potential. As prototypical endocrine-disrupting chemicals (EDCs), PFAS are linked to female reproductive impairment upon environmental exposure. Studies show elevated serum PFAS significantly raise ovarian dysfunction risk (diminished ovarian reserve, premature ovarian insufficiency).^1^ They also correlate with polycystic ovary syndrome (PCOS), endometriosis, and likely higher breast/ovarian cancer risks.^2^ Additionally, gestational PFAS exposure increases preeclampsia, gestational diabetes, and adverse outcomes (preterm delivery, spontaneous abortion, low birth weight).^3^

Endometriosis is a common inflammatory condition characterized by the presence of endometrium-like tissue outside the uterine cavity.^4^ It affects up to 10% of reproductive-aged women worldwide, including an estimated 9 million women in the United States.^5^ Endometriosis significantly impacts female fertility, potentially leading to infertility, miscarriage, and other complications.^6^^,^^7^ Additionally, Endometriosis is associated with an increased risk of premature death due to tumors, and the co-occurrence of endometriosis and uterine fibroids further elevates the risk of early mortality from cardiovascular diseases.^8^ The etiology and pathogenesis of endometriosis remain unclear, involving complex interactions among endocrine, immune, inflammatory, and environmental factors.^9^^,^^10^ For instance, one analysis of 33 PFAS compounds demonstrated that both individual substances (such as perfluorooctanoic acid (PFOA), perfluorooctanesulfonic acid (PFOS), and their isomers) and their mixtures were significantly associated with an increased risk of the disease.^11^ Similarly, another investigation found that higher serum levels of PFOA, PFOS, and perfluorononanoic acid (PFNA) were associated with an elevated risk.^12^ Further research not only confirmed the effects of individual substances such as perfluorotridecanoic acid (PFTrDA) but also, through mixture analysis, identified PFNA, perfluorohexanesulfonic acid (PFHxS), and PFTrDA as key drivers of the observed associations.^13^ Furthermore, an earlier study provided evidence for a positive association between both PFOA and PFNA and endometriosis risk.^14^ However, other studies have not observed significant associations. One cohort study reported no significant increase in endometriosis risk among women in the high PFAS exposure group.^15^ Similarly, a French study analyzing 14 PFAS compounds found no significant association between any individual PFAS and endometriosis risk.^10^ Therefore, further research is needed to clarify the relationship between PFAS and endometriosis.

Due to the complex etiology and incompletely elucidated pathological mechanisms of endometriosis, current treatments primarily focus on managing symptoms and improving the quality of life through medication and surgery, but there is no definitive cure.^16^ Investigating the processes, effects, thresholds, and mechanisms by which chemical substances induce endometriosis is of significant clinical and societal importance for pollutant control, understanding toxicity mechanisms, and developing interventions and treatments. Currently, research on PFAS and endometriosis remains largely confined to population-based associations, with studies on their toxicological mechanisms still lacking systematic depth. Network Toxicological Analysis serves as an analytical approach capable of rapidly and comprehensively revealing the complex molecular relationships between toxicants and pathologies,^17^ thereby providing an effective tool for assessing the toxicity and molecular biological mechanisms of environmental pollutants.^18^

This study utilizes data from the National Health and Nutrition Examination Survey (NHANES) and employs multiple statistical models to systematically evaluate the association between mixed PFAS exposure and the risk of endometriosis. Building on this, network toxicology approaches are applied to identify key biomolecules and pathways involved in PFAS-induced endometriosis, thereby establishing a complete evidence chain from exposure assessment to pathogenic mechanisms. This multi-level research strategy not only significantly enhances the body of evidence regarding health risks from mixed PFAS exposure but also provides new scientific insights for elucidating the molecular mechanisms of the disease and developing targeted prevention and treatment strategies.

## Results

### Characteristics of the study population

The characteristics of the participants are shown in Table 1. The study included 1,069 participants with a median age (P25, P75) of 35.9 (27, 44) years. The majority of participants were Mexican American (23.9%). The highest level of education attained by most participants was “some college or AA degree” (34.0%). Seventy-nine out of 1,069 women reported endometriosis, yielding a prevalence of 7.4% in this sample. Tobacco exposure was reported in 29.37% of participants, while 39.76% reported alcohol exposure.

**Table 1:** Characteristics of study participants

Participants characteristics	Mean (SD) or n (%)
Age	35.9 (10.0)
Race	
Mexican American	255 (23.9%)
Non-Hispanic Black	224 (21.0%)
Non-Hispanic White	505 (47.2%)
Other Hispanic	52 (4.9%)
Other Race	33 (3.1%)
BMI, kg/m^2^	29.4 (7.56)
PIR	2.62 (1.63)
Education	
9-11th grade	148 (13.8%)
College graduate or above	232 (21.7%)
High school graduate	230 (21.5%)
Less than 9th grade	96 (9.0%)
Some college or AA degree	363 (34.0%)
Tobacco_exposure	
No	502 (47.0%)
Yes	567 (53.0%)
Alcohol	
No	425 (39.8%)
Yes	644 (60.2%)
PFOA, ng/mL	3.80 (5.24)
PFOS, ng/mL	18.9 (19.1)
PFHxS, ng/mL	1.79 (2.06)
PFNA, ng/mL	0.963 (0.936)
Endometriosis	
No	990 (92.6%)
Yes	79 (7.4%)

### Results of logistic regression analysis

The results of the multivariate logistic regression are presented in Table 2. After adjusting for covariates such as age, ethnicity, and BMI, exposure to PFOA and PFOS significantly increased the risk of endometriosis, while PFHxS exposure showed a marginally significant association with endometriosis risk (*p* < 0.10).

**Table 2:** Logistic regression analysis

	PFAS	Model 1		Model 2^a^	
		OR (95%CI)	P-value	OR (95%CI)	P-value
Endometriosis	ln_PFOA	1.24(0.87,1.80)	0.242	1.63(1.17,2.28)	0.004
	ln_PFOS	1.38(0.97,1.98)	0.075	1.70(1.23,2.37)	0.001
	ln_PFHxS	1.13(0.88,1.46)	0.351	1.24(0.98,1.58)	0.074
	ln_PFNA	0.90(0.64,1.27)	0.551	1.18(0.88,1.58)	0.274

a Model 2 has adjusted for age and race, BMI, Educational level, tobacco exposure, alcohol exposure, and poverty ratio.

### Per- and polyfluoroalkyl substances mixed exposure and endometriosis risk: weighted quantile sum and Bayesian kernel machine regression analyses

**Figure 1:**
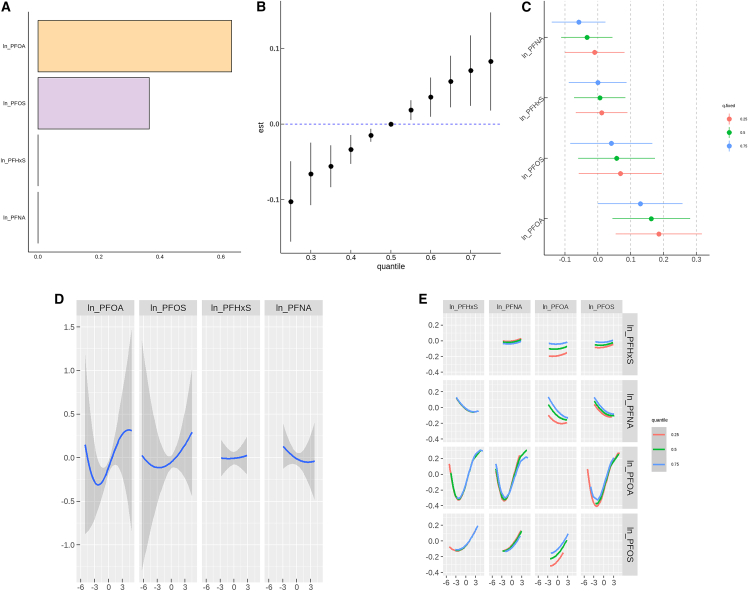
PFAS mixed exposure and endometriosis risk: WQS regression and BKMR results (A) WQS regression results. (B) Overall effect by the BKMR model (data points represent effect estimates (est), and error bars represent 95% confidence intervals (95% CI)). (C) Single-pollutant independent effect by the BKMR model (data points represent effect estimates of single pollutants under different fixed quantiles, and error bars represent 95% credible intervals). (D) Single-variable exposure-response relationships by the BKMR model. (E) Bivariate interaction analysis by the BKMR model.

The WQS regression showed statistically significant associations with endometriosis. After adjusting for covariates, the WQS index was positively associated with endometriosis (OR = 1.22, 95% CI: 1.08–1.39). Figure 1A illustrates the weights of individual PFAS compounds on the risk of endometriosis. PFOA had the highest weight for endometriosis risk (0.63), followed by PFOS (0.37).

To address the limitations of linearity and interactions in the previous analyses, BKMR was used to further evaluate the effects of PFAS mixtures on endometriosis. The BKMR results were consistent with the WQS findings. Figure 1B shows the overall effect of PFAS mixtures and endometriosis. Compared to the 50th percentile, the risk of endometriosis significantly increased when all PFAS levels were at or above the 50th percentile (P50). The importance of individual PFAS compounds, as indicated by their PIP, was ranked as follows: PFOA (PIP = 0.5880), PFOS (PIP = 0.2306), PFNA (PIP = 0.1504), and PFHxS (PIP = 0.0582).

In the single-pollutant independent effect BKMR model, PFOA, PFOS, and PFHxS were positively associated with endometriosis. When PFOA and other PFAS were at the P50, the risk of endometriosis increased by 18% (OR = 1.18, 95% CI: 1.05–1.33) (Figure 1C).

In the single-variable exposure-response relationships, the risk of endometriosis significantly increased when PFOA and PFOS exposures exceeded the P50, while the risk gradually decreased with PFNA exposure above P50 (Figure 1D). In the bivariate interaction analysis, no significant interactions were observed among the four PFAS compounds in relation to the risk of endometriosis (Figure 1E).

### Network toxicology analysis: Gene interaction networks/functional enrichment of per- and polyfluoroalkyl substances-endometriosis shared genes

**Figure 2:**
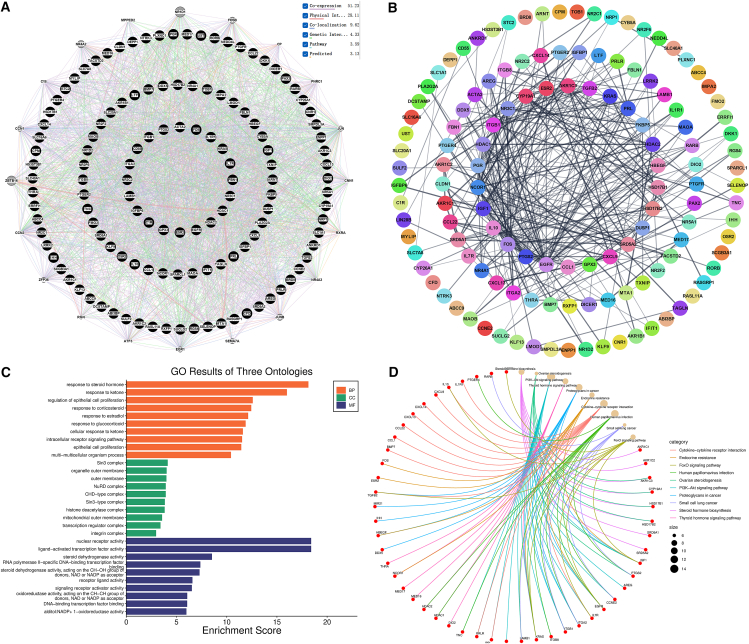
Network toxicology analysis: gene interaction networks and functional enrichment (A) GeneMANIA network of shared genes across PFAS and endometriosis. (B) PPI network of shared genes across PFAS and endometriosis. (C) The top ten enriched GO terms in each category. (D) The top ten KEGG pathways with the highest gene enrichment.

Using the CTD, 129 genes associated with both PFAS exposure and endometriosis were identified. The interaction patterns of these genes were further explored using GeneMANIA. As shown in Figure 2A, the primary type of interaction was co-expression (51.23%). Other types of gene-gene interactions included physical interactions (28.11%), co-localization (9.63%), genetic interactions (4.33%), pathway interactions (3.59%), and predicted interactions (3.13%).

To identify key proteins, a PPI network was constructed to illustrate the relationships and functions of proteins. The network consisted of 128 nodes and 305 edges (Figure 2B). Highly connected regions in the PPI network are more likely to be involved in biological regulation.

To investigate the potential mechanisms by which these four PFAS compounds induce endometriosis, GO and KEGG pathway enrichment analyses were performed on the identified genes. Figure 2C shows the top 10 enriched GO terms in each category. In the biological process (BP) category, the main enriched terms were response to steroid hormone, response to ketone, regulation of epithelial cell proliferation, response to corticosteroid, response to estradiol, response to glucocorticoid, cellular response to ketone, intracellular receptor signaling pathway, epithelial cell proliferation, and multicellular organism process. In the cellular component (CC) category, the main enriched terms were Sin3 complex, organelle outer membrane, outer membrane, NuRD complex, CHD-type complex, Sin3-type complex, histone deacetylase complex, mitochondrial outer membrane, transcription regulator complex, and integrin complex. In the molecular function (MF) category, the main enriched terms were nuclear receptor activity, ligand-activated transcription factor activity, steroid dehydrogenase activity, RNA polymerase II-specific DNA-binding transcription factor binding, steroid dehydrogenase activity (acting on the CH-OH group of donors, NAD or NADP as acceptor), receptor ligand activity, signaling receptor activator activity, oxidoreductase activity (acting on the CH-CH group of donors, NAD or NADP as acceptor), DNA-binding transcription factor binding, and alditol:NADP+ 1-oxidoreductase activity. The KEGG pathway enrichment analysis results are shown in Figure 2D. The significantly enriched pathways included steroid hormone biosynthesis, ovarian steroidogenesis, PI3K-Akt signaling pathway, thyroid hormone signaling pathway, proteoglycans in cancer, endocrine resistance, cytokine-cytokine receptor interaction, and FoxO signaling pathway.

### Hub gene identification via multiple algorithms

**Figure 3:**
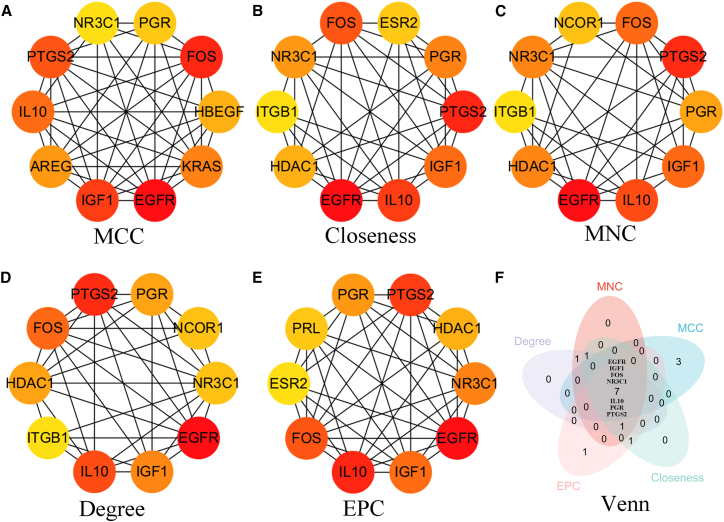
Key hub genes identified by multiple algorithms (A) Maximum clique centrality. (B) Closeness centrality. (C) Neighborhood component centrality. (D) Degree centrality. (E) Edge percolated component. (F) Venn diagram of 5 algorithm results.

Using the CytoHubba plugin in Cytoscape, the genes in the PPI network were ranked to identify key hub genes. The key hub genes were EGFR, FOS, IGF1, PTGS2, IL10, PGR, and NR3C1 (Figure 3).

### Hub gene tissue-specific expression and diagnostic value

**Figure 4:**
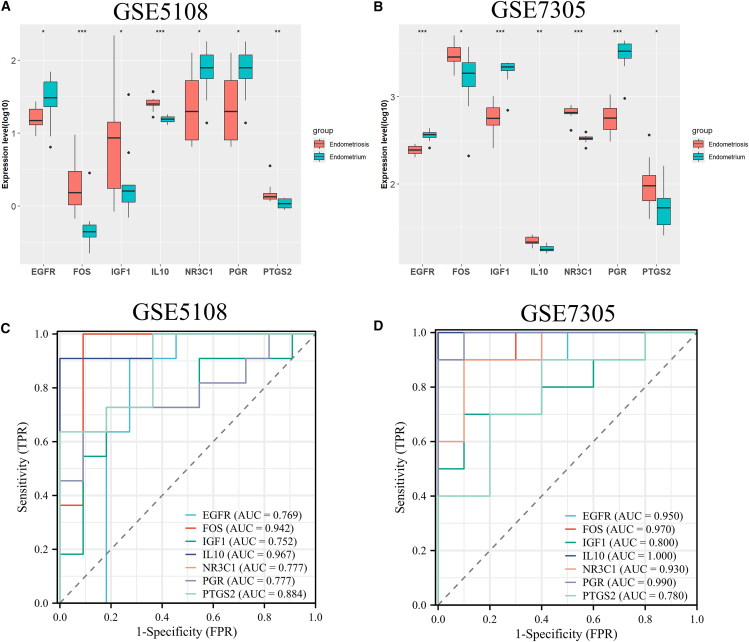
Hub gene tissue-specific expression and diagnostic value in endometriosis (A and B) Expression of seven key targets in endometriosis tissue and normal endometrial tissue (The box represents the interquartile range (IQR), spanning from the 25th percentile (Q1) to the 75th percentile (Q3); the horizontal line inside the box is the median (50th percentile). The whiskers extend to the maximum and minimum values within the ranges of Q1 - 1.5×IQR and Q3 + 1.5×IQR, respectively. Points beyond the whiskers are outliers). (C and D) The diagnostic effect of seven core targets in endometriosis tissue and normal endometrial tissue was evaluated using the subject work characteristic (ROC) curve.

Using data from GEO, we found that in different studies, the expression of these hub genes in endometriosis tissue was different from that in normal endometrial tissue (Figures 4A and 4B). We plotted the receiver operating characteristic (ROC) curve and calculated the area under the curve (AUC) to evaluate the diagnostic performance of these 7 core genes. The results showed that these 7 core targets have potential in the pathogenesis and future diagnosis of endometriosis (Figures 4C and 4D).

### Molecular docking: binding affinity of per- and polyfluoroalkyl substances with hub proteins

**Figure 5:**
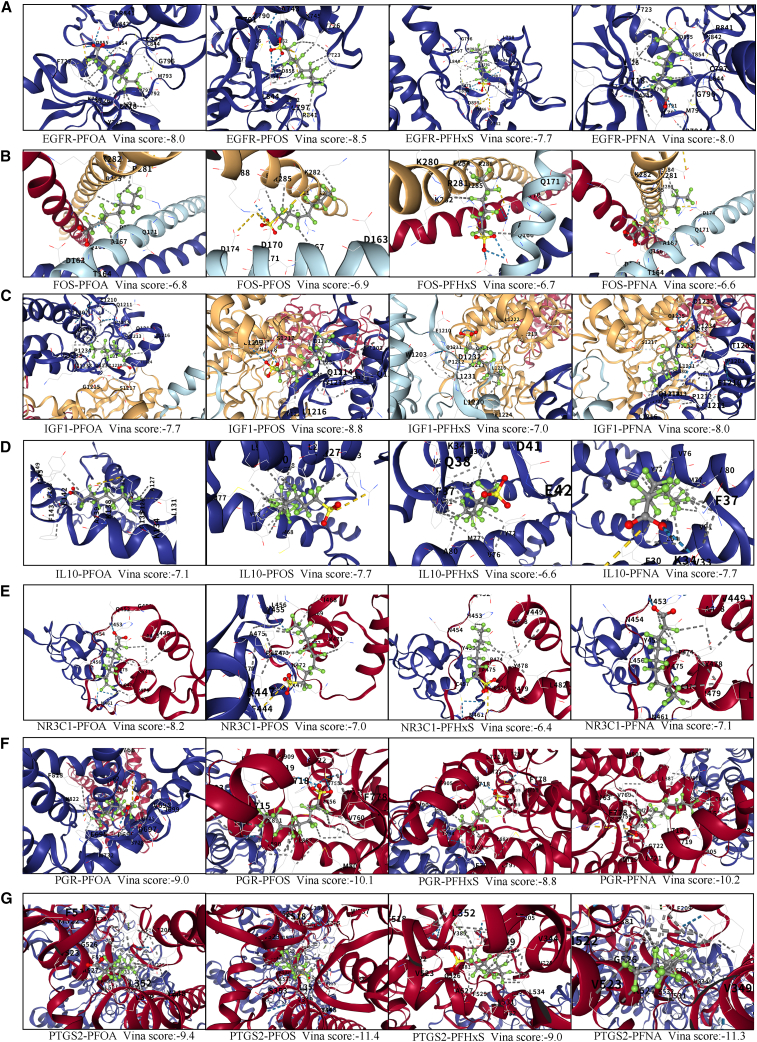
Binding affinity of PFAS with hub proteins: molecular docking results (A) EGFR and PFAS. (B) IGF1 and PFAS. (C) FOS and PFAS. (D) NR3C1 and PFAS. (E) IL10 and PFAS. (F) PGR and PFAS. (G) PTGS2 and PFAS.

We performed molecular docking analysis to assess interactions between PFOA, PFOS, PFHxS, and PFNA with seven core targets. PFOA, PFOS, PFHxS, and PFNA showed interaction with all seven targets. This indicates that PFAS has a strong binding affinity for these seven proteins, as the Vina scores are all below −5.^19^ Vina score and binding patterns are shown in Figure 5.

## Discussion

This study systematically investigated the association between mixed exposure to PFAS and the risk of endometriosis by integrating data from the NHANES with network toxicology approaches. The findings preliminarily revealed the potential molecular mechanisms by which PFAS contribute to the pathogenesis of endometriosis. The results demonstrated that mixed PFAS exposure was significantly positively associated with the risk of endometriosis, with specific PFAS compounds such as PFOA, PFOS, and PFNA playing critical roles in disease development. Additionally, we found that PFAS may participate in the pathogenesis of endometriosis through mechanisms such as lipid metabolism disruption, dysregulation of steroid hormone signaling pathways, and activation of the PI3K-Akt pathway. These findings not only provide important epidemiological evidence for the uterine toxicity of PFAS but also lay the groundwork for future environmental health policies and targeted intervention strategies.

### Per- and polyfluoroalkyl substances mixed exposure and the risk of endometriosis

Through weighted quantile sum (WQS) and Bayesian kernel machine regression (BKMR) analyses, we consistently found that exposure to mixtures of per- and polyfluoroalkyl substances (PFASs) was positively associated with the risk of endometriosis, with perfluorooctanoic acid (PFOA) and perfluorooctane sulfonate (PFOS) being the primary contributing factors (WQS weights: 0.63 and 0.37, respectively). This result is consistent with the study by Ao et al.,^11^ who reported a positive association between PFAS mixtures and endometriosis in a Chinese case-control study (adjusted odds ratio [aOR] = 1.24, 95% confidence interval [CI]: 1.05–1.48). However, it contradicts the findings of Hammarstrand et al.^15^ (a Swedish cohort study, hazard ratio [HR] = 0.74, 95% CI: 0.42–1.29) and Matta et al.^10^ (a French case-control study, with no significant association even after data pooling).

These discrepancies may stem from differences in study design across dimensions: types of PFAS (we focused on legacy PFAS including PFOA, PFOS, PFNA, and PFHxS; Ao et al. measured 33 types of PFAS, among which the branched isomer 1m-PFOS was the key driver [aOR = 1.16, posterior inclusion probability = 0.992]^11^; Hammarstrand et al. only tested 2 types of legacy PFAS, excluding PFOA and its isomers^15^; Matta et al. measured 14 types of PFAS, all at background exposure levels [PFOS median = 6 ng/mL]^10^); sample size (Ao et al.’s 574 participants balanced statistical power and diagnostic rigor^11^; Hammarstrand et al.’s large cohort had low case density^15^; Matta et al.’s small sample size lacked sufficient power for PFAS analysis^10^); study design (our study, along with Ao et al.’s and Matta et al.’s,^10^^,^^11^ used direct *in vivo* PFAS measurements; Hammarstrand et al. used indirect exposure assessment linked to residential addresses^15^); effect size (determined by PFAS toxicity and exposure levels); participant characteristics (variations in age, BMI, and socioeconomic status); adjustment for confounding factors (Ao et al. conducted comprehensive adjustments^11^; Hammarstrand et al. and Matta et al. omitted key confounders such as diet and hormone use^10^^,^^15^); and diagnostic methods (cases in Ao et al.’s and Matta et al.’s studies were confirmed by laparoscopy^10^^,^^11^; our study confirmed endometriosis diagnosis through participant self-report; Hammarstrand et al. relied on national registry data^15^; and controls in Matta et al.’s study lacked surgical confirmation^10^). In summary, the core causes of these discrepancies lie in the types and exposure levels of PFAS, the use of direct vs. indirect exposure assessment methods, and the rigor of diagnosis. Future studies should include legacy, emerging, and isomeric PFAS, adopt direct *in vivo* exposure measurements, confirm endometriosis via laparoscopy, and adjust for key confounders such as BMI, diet, and hormone use to clarify the association between PFAS and endometriosis.

The BKMR model further revealed a nonlinear relationship between mixed PFAS exposure and the risk of endometriosis. When all PFAS exposure levels exceeded the median, the risk of endometriosis significantly increased (OR = 1.18). This suggests a potential synergistic effect among PFAS compounds in the pathogenesis of endometriosis. These findings underscore the importance of considering the mixed effects of environmental pollutants, as traditional single-pollutant models may underestimate the cumulative impact of chemical exposures.

### Molecular mechanisms of per- and polyfluoroalkyl substance-induced endometriosis

Through network toxicology analysis, we identified 129 common genes associated with PFAS exposure and endometriosis. These genes were significantly enriched in steroid hormone responses (e.g., response to estradiol), inflammatory pathways (e.g., IL10, PTGS2), and cell proliferation regulation (e.g., EGFR). The identification of key hub genes, such as EGFR, FOS, and PTGS2, suggests that PFAS may contribute to the pathogenesis of endometriosis by disrupting hormone receptor signaling (e.g., nuclear receptor activity), activating oxidative stress responses (e.g., NADPH oxidase activity), and promoting abnormal epithelial cell proliferation. These findings align with the known endocrine-disrupting properties of PFAS,^20^^,^^21^ which have been shown to interfere with estrogen and progesterone signaling, leading to dysregulated proliferation and differentiation of endometrial cells.^22^

KEGG pathway enrichment analysis further indicated that PFAS may mediate the imbalance between endometrial cell survival and apoptosis through the PI3K-Akt and FoxO signaling pathways. This is consistent with previous studies demonstrating that environmental pollutants can induce endometriosis by disrupting these critical pathways.^23^ For instance, the PI3K-Akt pathway is often hyperactivated in endometriosis due to PTEN mutations or epigenetic silencing. PFAS may enhance EGFR phosphorylation, promote downstream Akt signaling, inhibit apoptosis, and increase the invasive potential of ectopic endometrial cells. Additionally, the interaction between PFAS and insulin-like growth factor 1 (IGF1) may exacerbate the metabolic-inflammatory-proliferative vicious cycle, further driving the progression of endometriosis.

Subsequently, we identified seven hub genes through multiple algorithms and confirmed their potential interaction with PFAS using molecular docking. In addition, data from two different studies suggest that these genes are differently expressed in endometriosis tissues than in normal tissues. The results of the ROC curve suggest that these core targets have potential value in the pathogenesis and future diagnosis of endometriosis. This suggests that these genes may play an important role in PFAS-induced endometriosis.

### Lipid metabolism dysregulation and inflammatory microenvironment

Lipid metabolism dysregulation is a hallmark of endometriosis. Our study suggests that PFAS exposure may disrupt the expression of apolipoproteins (e.g., APOE), impair high-density lipoprotein (HDL) function, and hinder cholesterol reverse transport, leading to intracellular lipid accumulation.^24^ Lipid accumulation can promote oxidative stress and inflammatory responses, activating pro-inflammatory pathways such as NF-κB, thereby creating a microenvironment conducive to the adhesion and invasion of ectopic endometrial cells.^25^ Furthermore, PFAS-induced lipid peroxidation products may directly damage endometrial cells,^26^ exacerbating tissue repair abnormalities.

### Dysregulation of steroid hormone signaling pathways

The significant enrichment of “steroid hormone biosynthesis” and “ovarian steroidogenesis” pathways in KEGG analysis aligns with the endocrine-disrupting properties of PFAS. Key genes in the PPI network, such as PGR and NR3C1, are core regulators of steroid hormone signaling.^27^ PFAS may mimic estrogen or antagonize progesterone receptors, disrupting the estrogen-progesterone balance and leading to uncontrolled endometrial cell proliferation.^28^ Additionally, PFAS may interfere with the secretion of FSH and LH, impairing ovarian function and promoting the development of endometriosis.^29^

### PI3K-Akt pathway activation and cell survival

The PI3K-Akt pathway is frequently hyperactivated in endometriosis due to PTEN mutations or epigenetic silencing. In this study, hub genes such as EGFR and IGF1 were identified as upstream regulators of this pathway. PFAS may enhance EGFR phosphorylation, promote Akt signaling, inhibit apoptosis, and increase the invasive capacity of ectopic endometrial cells.^30^ Moreover, the interaction between PFAS and IGF1 may further exacerbate the metabolic-inflammatory-proliferative cycle, driving the progression of endometriosis.^31^

### Immune regulation imbalance

The enrichment of genes such as IL10 and HBEGF in network toxicology analysis suggests that PFAS may contribute to endometriosis by disrupting immune tolerance mechanisms. IL10, an important anti-inflammatory cytokine, may exhibit abnormal expression, leading to macrophage polarization imbalance (M1/M2 ratio dysregulation),^29^ which prevents the immune system from effectively clearing refluxed endometrial fragments.^32^ Additionally, HBEGF (heparin-binding epidermal growth factor) may promote the proliferation and invasion of ectopic lesions through paracrine effects,^33^ a process that may be enhanced by PFAS exposure.

### Oxidative stress and the pathogenesis of endometriosis

Oxidative stress plays a critical role in the pathogenesis of endometriosis. Our study indicates that PFAS may regulate the expression of oxidative stress-related genes, such as NR3C1 and PGR, disrupting the normal function of endometrial cells and promoting the development of endometriosis. NR3C1, encoding the glucocorticoid receptor, is associated with enhanced oxidative stress responses in endometriosis.^34^ PGR, a key hormone receptor in endometriosis, regulates cell proliferation and apoptosis, contributing to disease progression.^28^ Furthermore, PFAS may impair the activity of antioxidant enzymes, leading to the accumulation of reactive oxygen species (ROS) in endometrial cells,^35^ thereby triggering oxidative stress and advancing endometriosis.

### Limitations of the study

This study integrated NHANES population data with molecular network analyses (from CTD, STRING, and so forth) to link PFAS exposure with endometriosis, though its findings are constrained by notable limitations that warrant emphasis. First, the limited number of endometriosis cases (*n* = 79) may reduce the statistical power of the analysis, diminishing the persuasiveness of the observed associations and necessitating caution in the interpretation of the results. Second, the diagnosis of endometriosis relied on self-reported information from participants. Such retrospective data are susceptible to recall bias, which may affect the accuracy of disease ascertainment. Furthermore, this study included prevalent cases, making it impossible to establish the temporal sequence between PFAS exposure and disease onset, thereby limiting causal inference. Third, although serum PFAS concentrations are a commonly used and relatively optimal method for assessing internal exposure, they may not fully reflect long-term exposure patterns. Fourth, due to limitations in the database, this study could not incorporate and adjust for important reproductive covariates such as parity, contraceptive use, and menstrual cycle characteristics. The absence of these potential confounding factors may have influenced the observed associations. Finally, only four common PFAS compounds were analyzed, primarily due to the low detection rates of other newly introduced PFAS in the samples, which hindered statistically robust analysis and thus limited the inclusion of newer PFAS compounds. Additionally, the network toxicology analysis was based on existing databases and computational algorithms; while it provides valuable mechanistic insights, further experimental validation is required to confirm the potential pathways. To address these limitations, future research should focus on targeted improvements, such as longitudinal designs and expanded experimental validation.

## Resource availability

### Lead contact

Requests for further information and resources should be directed to and will be fulfilled by the lead contact, Yuan Qian (yuanqian201601387@163.com).

### Materials availability

This study did not generate new unique reagents.

### Data and code availability

• Data: Human exposure and health data: from the National Health and Nutrition Examination Survey (NHANES) cycles 1999–2000 and 2003–2006, available via the National Center for Health Statistics website (https://www.cdc.gov/nchs/nhanes/); gene data associated with PFAS and endometriosis: from the Comparative Toxicogenomics Database (CTD, http://ctdbase.org/) with a screening threshold of p < 0.01; gene expression data of endometriosis: from the Gene Expression Omnibus (GEO, https://www.ncbi.nlm.nih.gov/geo/) with accession codes GSE5108 and GSE7305; protein structure data for molecular docking: from the Protein DataBank (PDB, https://www.rcsb.org/) with specific accession codes as follows: EGFR (4WKQ), FOS (1fos), IGF1 (1GZR), PTGS2 (1V0x), IL10 (1ILK), PGR (3ERT), and NR3C1 (1M2Z); and structural data of PFAS compounds: from the PubChem database (https://pubchem.ncbi.nlm.nih.gov/). CAS numbers are provided in the “key resources table” (PFOA: 335-67-1; PFOS: 1763-23-1; PFHxS: 355-46-4; PFNA: 375-95-1).
• Code: No original code was generated in this study. Software and R packages used for data analysis are as follows: R 4.1.1 (https://www.r-project.org/), glmnet package, gWQS package, bkmr package, and clusterProfiler package. Relevant analysis parameters are detailed in the “STAR Methods” section.
• All other items: All additional information required to reproduce the results of this study can be obtained from the corresponding author.

## Acknowledgments

This study was supported by the Dongguan Social Science and Technology development project (Grant No. 20211800901682).

## Author contributions

Conceptualization, H.Z., S.Z., Y.L., W.H., and Q.Y.; methodology, H.Z., W.H., and Y.L.; data curation, H.Z., J.Y., and F.Z.; formal analysis, F.Z.; visualization, J.Y.; investigation, X.Y.; validation, X.Y.; resources, W.H. and Y.L.; project administration, Y.L.; supervision, Y.L., W.H., and Q.Y.; writing – original draft, H.Z. and S.Z.; writing – review and editing, Q.Y.

## Declaration of interests

The authors declare no competing interests.

## STAR★Methods

### Key resources table

**Table:** 

RESOURCE or REAGENT	SOURCE	IDENTIFIER
**Chemical, peptide, or recombinant protein**s		

Perfluorooctanoic acid (PFOA)	PubChem	CAS: 335-67-1
Perfluorooctane sulfonic acid (PFOS)	PubChem	CAS: 1763-23-1
Perfluorohexane sulfonic acid (PFHxS)	PubChem	CAS: 355-46-4
Perfluorononanoic acid (PFNA)	PubChem	CAS: 375-95-1
EGFR	Protein DataBank (PDB)	4WKQ
FOS	Protein DataBank (PDB)	1fos
IGF1	Protein DataBank (PDB)	1GZR
PTGS2	Protein DataBank (PDB)	1V0x
IL10	Protein DataBank (PDB)	1ILK
NR3C1	Protein DataBank (PDB)	1M2Z
PGR	Protein DataBank (PDB)	3ERT

**Critical commercial assays**		

Online SPE-HPLC-ID-MS/MS Method	Kuklenyik et al.^36^	https://doi.org/10.1021/ac050671l

**Deposited data**		

NHANES 1999–2000,2003-2006 Data	National Center for Health Statistics	https://www.cdc.gov/nchs/nhanes/
GEO: GSE5108	Gene Expression Omnibus	GEO: GSE5108
GEO: GSE7305	Gene Expression Omnibus	GEO: GSE7305

**Software and algorithms**		

R 4.1.1	R oundation	https://www.r-project.org/
glmnet package	Friedman et al.^37^	https://doi.org/10.18637/jss.v033.i01
gWQS package	Gennings et al.^38^	https://doi.org/10.1016/j.mex.2025.103580
bkmr package	Bobb et al.^39^	https://doi.org/10.1093/biostatistics/kxu058
clusterProfiler package	Yu et al.^40^	https://doi.org/10.1089/omi.2011.0118
STRING Database	Szklarczyk et al.^41^	https://string-db.org/
Cytoscape	Shannon et al.^42^	https://doi.org/10.1101/gr.1239303
CytoHubba Plugin	Chin et al.^43^	https://doi.org/10.1186/1752-0509-8-S4-S11
CB-Dock2	Liu et al.^44^	https://doi.org/10.1093/nar/gkac394

**Other**		

Comparative Toxicogenomics Database (CTD)	Davis et al.^45^	http://ctdbase.org/
GeneMANIA	Warde-Farley et al.^46^	https://genemania.org/
PubChem Database	Kim et al.^47^	https://pubchem.ncbi.nlm.nih.gov/
Protein DataBank (PDB)	Berman et al.^48^	https://www.rcsb.org/

### Experimental model and study participant details

#### Human study participants

This study utilized de-identified, publicly available data from the National Health and Nutrition Examination Survey (NHANES) cycles 1999–2000, 2003–2004, and 2005–2006. The analysis was restricted to female participants aged 20–50 years who had available data on both self-reported endometriosis diagnosis and serum concentrations of per- and polyfluoroalkyl substances (PFAS). All NHANES protocols were approved by the National Center for Health Statistics Research Ethics Review Board, and informed consent was obtained from all participants. Given that the current analysis constitutes secondary examination of anonymized datasets in which personal identifiers had been permanently removed, obtaining additional institutional ethics approval was not mandated under prevailing regulatory provisions.

The following demographic and socioeconomic covariates were included: age (continuous), race/ethnicity (categorical), education level (categorical), poverty-to-income ratio (PIR, continuous), and body mass index (BMI, categorical). Sex and gender were self-reported as female. The influence of sex and gender on the study results is inherent to the study design, as the analysis was confined to females due to the sex-specific nature of endometriosis.

### Method details

#### PFAS quantification

Serum concentrations of twelve PFAS, including perfluorooctanoic acid (PFOA), perfluorooctane sulfonic acid (PFOS), perfluorononanoic acid (PFNA), and perfluorohexane sulfonic acid (PFHxS), were determined at the National Center for Environmental Health, Centers for Disease Control and Prevention (CDC). This method employs on-line solid-phase extraction coupled with high-performance liquid chromatography-isotope dilution tandem mass spectrometry (on-line SPE-HPLC-isotope dilution-tandem mass spectrometry, abbreviated as on-line SPE-HPLC-MS/MS). The detailed procedure is as follows: 100 μL of thawed serum is mixed with isotope-labeled internal standards (e.g., ^13^C_8_-PFOA, ^13^C_8_-PFOS, ^13^C_2_-PFHxS), filtered through a 0.2-μm protein precipitation plate, and then injected into an on-line SPE column (2.1 × 50 mm). After matrix removal with 0.1% acetic acid, the analytes are transferred to a Betasil C18 analytical column (2.1 × 50 mm, 3 μm), which is operated at 40°C. Separation is performed using a 7-min methanol/2 mM ammonium acetate gradient at a flow rate of 0.3 mL/min. Detection is carried out with a triple quadrupole mass spectrometer in negative electrospray ionization mode with selected reaction monitoring.

Quantification was achieved via a 1/x-weighted linear regression of the analyte-to-internal-standard peak-area ratio against a six-point calibration curve (0.05–200 ng/mL) prepared in human serum. Quality control pools (*n* = 40 per run) demonstrated inter-day coefficients of variation <15% and accuracies within ±10%. The limits of detection (LOD) ranged from 0.05 to 0.2 ng/mL. Values below the LOD were imputed as LOD/√2 for statistical analysis. PFOA, PFOS, PFHxS, and PFNA were detected in >90% of participants and were retained for primary analysis.

#### Covariate assessment

Covariates were obtained from NHANES questionnaires and examination data. Tobacco exposure was defined as a serum cotinine concentration >0.05 ng/mL. Alcohol use was defined as consuming more than one drink per week in the past 12 months. Detailed descriptions of all variables are available at the NHANES website: http://www.cdc.gov/nchs/nhanes/.

#### Gene identification

Genes associated with endometriosis and the four PFAS (PFOA, PFOS, PFHxS, PFNA) were retrieved from the Comparative Toxicogenomics Database (CTD) using the SetAnalyzer tool. A *p*-value threshold of 0.01 was applied.

#### Gene-gene interaction network

Protein-protein interaction networks among the identified genes were constructed using GeneMANIA (https://genemania.org) for Homo sapiens.

#### Functional enrichment analysis

Gene Ontology (GO) and Kyoto Encyclopedia of Genes and Genomes (KEGG) pathway enrichment analyses were performed using the “clusterProfiler” package in R.

#### Hub gene identification

A protein-protein interaction (PPI) network was built using the STRING database (organism: Homo sapiens; required confidence score: 0.700). Hub genes within the network were identified using the CytoHubba plugin in Cytoscape, employing five algorithms (MCC, Closeness, MNC, Degree, EPC).

#### Hub gene expression validation

Transcriptomic data from Gene Expression Omnibus (GEO) datasets GSE5108 and GSE7305, comprising 21 normal endometrial and 21 endometriosis tissue samples, were analyzed to compare the expression levels of the identified hub genes.

#### Molecular docking

The interactions between the four PFAS (ligands) and the hub gene-encoded proteins (receptors) were assessed by molecular docking using CB-Dock2. The SMILES structures of PFAS were obtained from PubChem, and the 3D structures of the target proteins were retrieved from the Protein DataBank (PDB). The docking pose with the lowest Vina score was selected as the optimal binding mode, and the binding interactions were visualized.

### Quantification and statistical analysis

The normality of continuous variables was assessed using the Kolmogorov-Smirnov test. Non-normally distributed variables (age, PIR) are summarized as median (interquartile range) and compared using the Mann-Whitney U test. Categorical variables are presented as frequencies (percentages) and compared using the Chi-squared test.

PFAS concentrations were natural-log-transformed to approximate a normal distribution. The associations between individual ln-transformed PFAS and endometriosis were evaluated using multivariable logistic regression models, adjusted for age, PIR, race/ethnicity, education, BMI, alcohol use, and tobacco exposure.

The combined effect of the PFAS mixture was assessed using weighted quantile sum (WQS) regression and Bayesian kernel machine regression (BKMR). For WQS regression, the data were randomly split into a training set (40%) for weight estimation and a validation set (60%) for inference. Deciles and 1,000 bootstrap samples were used, with a positive directional constraint. For BKMR, PFAS concentrations were standardized, and 10,000 Markov chain Monte Carlo iterations were run to estimate posterior inclusion probabilities. The following results are reported: the overall mixture effect, the effect of individual PFAS conditional on others fixed at specific percentiles, exposure-response functions, and pairwise interactions.

All statistical analyses were performed using R software (version 4.1.1) with the ‘glmnet’, ‘gWQS’, and ‘bkmr’ packages. A two-sided *p*-value <0.05 was considered statistically significant. The exact value of n (sample size) for each analysis is provided in the results section and figure legends. n represents the number of individual participants. Data are presented as mean ± standard deviation (SD) or median (IQR) as appropriate. Statistical tests were not blinded, and no data were excluded from the primary analyses. Sample size was determined by the available NHANES data; no *a priori* power calculation was performed.
